# Total 25-hydroxy vitamin D level in cerebrospinal fluid correlates with serum total, bioavailable, and free 25-hydroxy vitamin D levels in Korean population

**DOI:** 10.1371/journal.pone.0213389

**Published:** 2019-03-19

**Authors:** Dong-Hyun Lee, Jin Hyun Kim, Myeong Hee Jung, Min-Chul Cho

**Affiliations:** 1 Department of Laboratory Medicine, Gyeongsang National University Hospital, Gyeongsang National University College of Medicine, Jinju, Republic of Korea; 2 Biomedical Research Institute, Gyeongsang National University Hospital, Jinju, Republic of Korea; 3 Institute of Health Science, Gyeongsang National University, Jinju, Republic of Korea; Stockholm University, UNITED STATES

## Abstract

Epidemiological investigations have suggested that serum 25-hydroxyvitamin D [25(OH)D] level has significantly inverse associations with various neurological and neurodegenerative diseases. However, little is known about 25(OH)D level in human cerebrospinal fluid (CSF). Thus, the aim of this study was to determine correlations of 25(OH)D level in CSF with serum total, bioavailable, and free 25(OH)D levels. This observational study enrolled a total of 117 subjects (58 patients with non-neurologic disease and 59 patients with neurologic disease) from 2017 to 2018. CSF and blood samples were collected in pairs. Total 25(OH)D levels in CSF and serum and vitamin D binding protein (VDBP) levels in serum were measured. We also performed GC genotyping for polymorphisms of rs4588 and rs7041 to calculate bioavailable and free 25(OH)D levels. CSF total 25(OH)D levels were compared with serum total, bioavailable, and free 25(OH)D levels. Mean total 25(OH)D concentrations in CSF and serum of all patients were 37.14 ± 7.71 and 25.72 ± 12.37 ng/mL, respectively. The mean total 25(OH)D concentration in CSF was generally 1.4-fold higher than that in the serum. Total 25(OH)D concentrations in CSF showed weakly positive but significant correlations with serum total, bioavailable, and free 25(OH)D concentrations (*P* = 0.022, *P* = 0.033, and *P* = 0.026, respectively). Serum total 25(OH)D concentration was also correlated with serum VDBP concentration (*P* = 0.017). However, total 25(OH)D levels in CSF of non-neurologic disease group and neurologic disease group were similar. Total 25(OH)D level in CSF has weakly positive but significant correlations with serum total, bioavailable, and free 25-hydroxy vitamin D levels in the Korean population. The distribution of CSF total 25(OH)D in Korean neurologic and non-neurologic disease patients was presented.

## Introduction

Most vitamin D in humans are synthesized in the skin exposed to sunlight, although some minor portions of vitamin D can be acquired form the diet. Two steps of hydroxylation are required to obtain active form of vitamin D. The first hydroxylation occurs in the liver where vitamin D is converted to 25-hydroxy vitamin D [25(OH)D] which is recognized as the optimal indicator of vitamin D metabolic status due to its relatively long half-life and high concentration in the serum compared to other forms of vitamin D metabolites [1]. Then 25(OH)D is transported to kidneys where it is converted to 1α, 25-dihydroxy vitamin D [1α, 25(OH)_2_D], an active form of vitamin D [2,3]. This active form of vitamin D performs a variety of functions in tissues and organs throughout the body. Most (85%–90%) of circulating vitamin D are tightly bound to vitamin D-binding protein (VDBP), although a smaller amount (10%–15%) are loosely bound to albumin. Less than 1% of circulating vitamin D are present in free unbound form [4–6]. Since the affinity of 25(OH)D or 1α, 25(OH)_2_D to albumin is weaker than that to VDBP, the loosely binding fraction and the free fraction are considered bioavailable 25(OH)D [4].

VDBP, a 58-kDa multifunctional protein that is produced in the liver, circulates in the plasma. VDBP is an acute phase reactant. Its level can change depending on various conditions [7–10]. The gene encoding VDBP (*GC*) has a high rate of polymorphism. The frequency of its genotype is different depending on ethnic population. In addition, differences in VDBP affinity for 25(OH)D depending on the genotype have been reported. Two single-nucleotide polymorphisms (SNPs), rs7041 and rs4588, give rise to three major polymorphic isoforms of VDBP (GC1F, GC1S, and GC2). Their frequencies differ among ethnic populations. Isoform GC1F has the highest affinity for vitamin D, followed by GC1S and GC2 [11,12]. Since the dissociation constant of VDBP (i.e., affinity) is used in the calculation of bioavailable 25(OH)D [4,5], the level of bioavailable 25(OH)D is affected by serum VDBP level and *GC* genotype. Because the affinity of VDBP for 25(OH)D is dependent on the polymorphic isoform, it may be important to take GC genotype (rs7041 and rs4588) into account for accurate determination of bioavailable 25(OH)D [9,10,13].

Both vitamin D receptor and 1α-hydroxylase that catalyzes the formation of 1α, 25(OH)_2_D have been identified on neurons and oligodendrocytes of human and animal brains [14–16]. Moreover, 1α, 25(OH)_2_D can induce neuronal expression of nerve growth factor and other central nervous system (CNS) proteins [17]. Intrathecal vitamin D may therefore play a role in modulating inflammation and inducing regeneration of neuron.

Epidemiological investigations have suggested that serum 25-hydroxyvitamin D (25(OH)D) level has significantly inverse associations with various neurological and neurodegenerative diseases [18–23]. However, little is known about 25(OH)D level in human cerebrospinal fluid (CSF). In a previous study, vitamin D levels in CSF of patients with Alzheimer’s disease have been measured and compared with those of normal subjects [24]. A few animal injection studies have also compared serum vitamin D levels and CSF vitamin D levels [25,26]. However, studies measuring vitamin D concentration in human CSF specimens in comparison with serum vitamin D levels have not been reported yet. Thus, the objective of this study was to determine correlation of 25(OH)D level in CSF with serum 25(OH)D level. We present the first study comparing 25(OH)D level in CSF from 117 patients with total, bioavailable, and free 25(OH)D concentrations in serum considering VDBP genotypes in Koreans.

## Materials and methods

### Study subjects

This observational study enrolled a total of 117 subjects who underwent lumbar puncture for the purpose of diagnosis from August 2017 to June 2018. Patient's CSF and blood samples were collected in pairs on the same day. We collected clinical and laboratory data, including age, sex, albumin level, cellular differential count of CSF, chemical analysis of CSF including glucose and protein level, and final diagnosis from electronic medical records. These enrolled patients were classified into two groups (non-neurologic disease and neurologic disease groups) depending on the result of CSF analysis and the final diagnosis. If the result of CSF analysis was normal and the final diagnosis was not related to neurologic pathology, the patient was classified into the non-neurologic disease group.

When the number of red blood cells exceeded 1,000 in differential count for CSF, it was regarded as traumatic tapping and excluded from this study. At the time of study enrollment, CSF and blood samples were collected. Serum and leukocytes were then separated and stored at -80°C. The study protocol was approved by the Institutional Review Board of Gyeongsang National University Hospital (approval number: 2017-03-010). Written informed consent was obtained from each participant.

### VDBP and total 25(OH)D assays

For CSF sample, only total 25(OH)D level was measured. For serum sample, VDBP level was also measured to calculate bioavailable concentration with total 25(OH)D. VDBP was measured using an enzyme-linked immunosorbent assay (ELISA) kit (R&D Systems, Minneapolis, MN, USA) according to the manufacturer’s protocol. Total 25(OH)D was measured using Elecsys Vitamin D Total Electrochemiluminescence Binding Assay (Roche Diagnostics, Mannheim, Germany) with a Cobas 8000 e602 analyzer (Roche Diagnostics).

### GC genotyping

Genomic DNA was isolated from peripheral blood leukocytes using a DNeasy Blood and Tissue Kit (Qiagen, Hilden, Germany) according to the manufacturer’s instructions. *GC* genotyping for rs7041 and rs4588 was performed using a TaqMan SNP Genotyping Assay (Thermo Fisher Scientific, Waltham, MA, USA) and an ABI ViiA 7 Real-Time PCR System (Applied Biosystems, Foster City, CA, USA) according to manufacturers’ instructions. Common *GC* alleles were determined as follows: Gc1f (c.1296T; c.1307C), Gc1s (c.1296G; c.1307C), and Gc2 (c.1296T; c.1307A).

### Calculation of serum bioavailable and free 25(OH)D

Based on total 25(OH)D, VDBP, and albumin levels, serum bioavailable and free 25(OH)D levels were calculated using the following equations [13].

$$\begin{array}{l} Bioavailable\;25\left(OH\right)D=albumin‑bound\;25\left(OH\right)D+free\;25\left(OH\right)D\\ \;=\left(albumin\times K_{albumin}+1\right)\times free\;25\left(OH\right)D \end{array}$$

$$\mathrm{Free}\;25(\mathrm{OH})D=\frac{-b+\sqrt{b^{2}-4ac}}{2a}$$

a = *K*_*VDBP*_ × *K*_*albumin*_ × albumin + *K*_*VDBP*_

b = *K*_VDBP_ × VDBP - *K*_*VDBP*_ × total 25(OH)D + *K*_*albumin*_ × albumin + 1

c = - Total 25(OH)D

*K*_*albumin*_ = 6 × 10^5^ M^-1^

To calculate bioavailable 25(OH)D levels, variable *K*_*VDBP*_ was replaced by genotype-specific VDBP binding affinity (*K*_*VDBP1f*_, 1.12 × 10^9^ M^-1^; *K*_*VDBP1s*_, 0.6 × 10^9^ M^-1^; *K*_*VDBP2*_, 0.36 × 10^9^ M^-1^) [11]. For heterozygous genotypes, mean affinity for these two homozygotes was used (*K*_*VDBP1f/1s*_, 0.86 × 10^9^ M^-1^; *K*_*VDBP1f/2*_, 0.74 × 10^9^ M^-1^; *K*_*VDBP1s/2*_, 0.48 × 10^9^ M^-1^) [27].

### Statistical analysis

Frequencies are expressed as percentage (%) for categorical variables. Continuous variables are presented as median value and range. We compared vitamin D related data and *GC* genotypes between groups using Pearson’s Chi-square test for categorical variables. The significance of normally distributed variables was calculated by one-way analysis of variance (ANOVA) followed by post hoc Tukey’s test. The relationship between vitamin D related variables was evaluated by simple correlation analysis. The distribution of CSF total 25(OH)D was calculated as follows. After excluding outliers using the Tukey method, data set was subjected to nonparametric analysis (2.5–97.5th percentile interval). All statistical analyses were performed using PAWS Statistics software, version 18.0 (SPSS Inc., Chicago, IL, USA) and MedCalc Statistical software, version 17.2 (Mariakerke, Belgium). *P* values < 0.05 were considered statistically significant.

## Results

### General characteristics of study subjects

A total of 117 patients were enrolled in this study, including 53 (45.3%) males and 64 (54.7%) females. The male to female ratio was 0.83:1. The median age of these patients was 53 years (range, 24 days– 87 years). Among patients included in the study, the most frequent age group was 16 to 65 years (42.7%), followed by the age group of over 65 years (30.8%). Among 117 patients, 58 (49.6%) and 59 (50.4%) patients were classified into non-neurologic disease and neurologic disease groups, respectively. In the non-neurologic disease group, there were 27 (46.6%) patients with infectious disease, 17 (29.3%) with headache, 8 (13.8%) with fever, and 6 (10.3%) with sepsis. In the neurologic disease group, 17 (14.5%) patients had meningitis, including 3 with bacterial meningitis, 13 with viral meningitis, and 1 with tuberculosis meningitis. There were also 15 (12.8%) patients with traumatic neurologic disease such as subarachnoid hemorrhage (SAH) and 27 (23.1%) patients with other neurologic diseases, including tumors, motor neuron disease, hydrocephalus, and so on. General characteristics and demographics of study subjects are summarized in Table 1.

**Table 1:** Characteristics of patients enrolled in this study.

Characteristics	Total	Neurologic disease	Non-neurologic disease
Total number of patients	117	59	58
Sex ratio (Male:Female)	0.83 : 1 (53 : 64)	1.19 : 1 (32 : 27)	0.57 : 1 (21 : 37)
Age, median (IQR)	53 (17–69)	62 (51–73)	25 (2.5–54.5)
Height, median (Cm, IQR)	155 (142.3–164.5)	160 (152.2–168.0)	150 (94.7–162.1)
Weight, median (Kg, IQR)	54.6 (37.7–64.5)	57.9 (46–66)	49 (13.4–63)
Age, number of cases			
Under 1 year	15	2	13
1 year to 17 year	16	4	12
18 year to 65 year	50	28	22
Over 65 year	36	25	11
Disease, number of cases			
Infectious disease			27
Headache			17
Fever			8
Sepsis			6
Meningitis		17	
Bacterial meningitis		3	
Viral meningitis		13	
Tuberculosis meningitis		1	
Traumatic		15	
Subarachnoid hemorrhage		9	
Intracranial hemorrhage		5	
Epidural hematoma		1	
Others		27	
Tumor		7	
Motor neuron disease		5	
Hydrocephalus		3	
Seizure		3	
Encephalopathy		2	
Plexopathy		2	
Decreased mentality		1	
Febrile convulsion		1	
Headache syndrome		1	
Moyamoya syndrome		1	
Parkinson syndrome		1	

IQR = Interquartile Range

### Comparison of Vitamin D concentration between CSF and serum

Mean total 25(OH)D concentrations in CSF and serum of all patients were 37.14 ± 7.71 and 25.72 ± 12.37 ng/mL, respectively. The mean total 25(OH)D concentration in CSF was generally 1.4-fold higher than that in the serum. Mean serum bioavailable and free 25(OH)D levels in all patients were 4.01 ± 3.10 ng/mL and 11.15 ± 8.60 pg/mL, respectively (Table 2).

**Table 2:** Comparison of Vitamin D levels in serum and CSF by age groups.

Age group	CSF total 25(OH)D (ng/mL)	Serum total 25(OH)D (ng/mL)	Serum bioavailable 25(OH)D (ng/mL)	Serum free 25(OH)D (pg/mL)	Serum VDBP (ug/mL)
< 1 year	37.62 ± 7.97	38.37 ± 10.10	5.93 ± 3.17	15.90 ± 8.21	192.68 ± 52.11
1 ~ 17 years	40.56 ± 5.76	33.25 ± 13.53	5.30 ± 3.46	14.12 ± 10.52	187.01 ± 54.74
18 ~ 65 years	35.49 ± 8.36	20.20 ± 9.06	2.69 ± 1.66	7.32 ± 4.25	194.48 ± 49.01
>65 years	37.73 ± 7.08	24.77 ± 11.58	4.45 ± 3.72	13.18 ± 10.31	165.54 ± 61.37
Total	37.14 ± 7.71	25.72 ± 12.37	4.01 ± 3.10	11.15 ± 8.60	184.32 ± 55.05
*P* value	0.125	**<0.001**	**<0.001**	**<0.001**	0.097

Data are expressed as means ± SD. CSF = Cerebrospinal fluid; 25(OH)D = 25-hydroxyvitamin D.

When analyzing vitamin D and VDBP levels by age, CSF total 25(OH)D concentration was the highest in the age group of 1–17 years but the lowest in the age group of 18 to 65 years. Serum total, bioavailable, and free 25(OH)D levels were the lowest in the age group of 18–65 years but the highest in the 1-year old group. Differences in serum total, bioavailable, and free 25(OH)D levels by age were statistically significant (*P* < 0.001). Mean serum VDBP level in all patients was 184.32 ± 55.05 ug/mL. It showed no significant difference among age groups (Table 2).

When analyzing vitamin D and VDBP levels by disease, CSF total 25(OH)D concentration in the non-neurologic disease group was 37.78 ± 7.75 ng/mL. In the non-neurologic disease group, CSF and serum total 25(OH)D levels in sepsis patients showed the highest concentrations. However, they were not significantly different from those in other patients of the same non-neurologic disease group. For CSF total 25(OH)D concentration in the neurologic disease group, it was 37.94 ± 7.46 ng/mL in the sub-group of meningitis, 35.26 ± 8.64 ng/mL in the sub-group of traumatic disease, and 36.34 ± 7.44 ng/mL in the sub-group of others. Serum total 25(OH)D levels were lower in all sub-groups of the neurologic disease group than those in the non-neurologic disease group. Serum bioavailable and free 25(OH)D concentrations were the lowest in the sub-group of traumatic disease but the highest in the non-neurologic disease group. Serum VDBP level was the highest in the sub-group of traumatic disease followed by that in the sub-group of meningitis. However, there was no significant difference in all variables according to disease (Table 3).

**Table 3:** Comparison of vitamin D levels in serum and CSF by disease.

Disease group	CSF total 25(OH)D (ng/mL)	Serum total 25(OH)D (ng/mL)	Serum bioavailable 25(OH)D (ng/mL)	Serum free 25(OH)D (pg/mL)	Serum VDBP (ug/mL)
Non-neurologic disease	37.78±7.75	27.37±14.05	4.52±3.74	12.22±10.02	177.17±58.51
Infectious disease	38.41±7.19	27.24±14.94	4.14±3.18	11.14±7.88	179.88±60.04
Headache	36.55±9.39	26.36±13.00	5.20±4.79	13.69±12.53	161.39±61.13
Fever	35.55±8.47	21.01±13.06	4.12±4.50	12.21±14.37	173.04±50.56
Sepsis	41.39±1.59	39.32±8.42	4.78±1.45	12.96±3.91	215.15±45.81
Neurologic disease	36.52±7.69	24.10±10.32	3.50±2.23	10.10±6.84	191.36±50.94
Meningitis	37.94±7.46	24.79±11.40	3.57±2.17	10.62±6.94	199.29±35.81
Traumatic	35.26±8.64	21.86±7.78	2.81±2.07	8.50±6.02	205.00±34.93
Others	36.34±7.44	24.91±10.99	3.85±2.33	10.67±7.31	178.78±63.47
Total	37.14±7.71	25.72±12.37	4.01±3.10	11.15±8.60	184.32±55.05
*P* value*	0.639	0.099	0.172	0.474	0.760

Data are expressed as means ± SD. CSF = Cerebrospinal fluid; 25(OH)D = 25-hydroxyvitamin D.

* = compare with sub-groups of neurologic and non-neurologic disease using ANOVA.

### Vitamin D levels in CSF and serum by genotypes

Among six genotypes of vitamin D, Gc1s-1f type had the largest proportion (N = 45, 38.5%), followed by GC2-1f (N = 19, 16.2%) and Gc1f-1f (N = 18, 15.4%). Those with Gc1s-1s genotype had the highest concentrations of CSF total 25(OH)D. Serum total 25(OH)D and serum VDBP concentrations were the highest in those with Gc2-1s genotype but the lowest in those with Gc2-2 genotype. Serum bioavailable 25(OH)D concentration was the highest in those with Gc2-1s genotype (6.41 ± 3.65 ng/mL) but the lowest in those with Gc1f-1f genotype (2.09 ± 0.94 ng/mL). Serum free 25(OH)D concentration was also the highest in the Gc2-1s group (18.19 ± 10.74 pg/mL) but the lowest in the Gc1f-1f group (5.90 ± 2.47 pg/mL). Serum bioavailable and serum free 25(OH)D concentrations showed significant differences according to genotype (*P* = 0.001 and *P* < 0.001, respectively) (Table 4). However, there was no significant difference in genotype distribution between the non-neurologic disease group and the neurologic disease group (*P* = 0.171).

**Table 4:** Comparison of vitamin D levels in serum and CSF by genotypes.

Genotype group	CSF total 25(OH)D (ng/mL)	Serum total 25(OH)D (ng/mL)	Serum bioavailable 25(OH)D (ng/mL)	Serum free 25(OH)D (pg/mL)	Serum VDBP (ug/mL)
Gc1f-1f (N = 18)	36.06±8.98	22.6±11.65	2.09±0.94	5.90±2.47	184.71±69.89
Gc1s-1f (N = 45)	35.92±8.62	25.39±11.35	3.46±3.22	9.40±8.90	191.56±53.58
Gc1s-1s (N = 9)	40.93±2.22	26.37±12.80	3.88±1.74	11.50±5.47	188.00±45.08
Gc2-1f (N = 19)	38.59±7.15	26.48±13.84	4.43±2.77	12.09±7.17	162.57±49.10
Gc2-1s (N = 14)	36.27±8.29	32.36±14.02	6.41±3.65	18.19±10.74	196.12±60.15
Gc2-2 (N = 12)	39.26±2.79	22.14±11.63	5.53±3.24	15.66±8.10	174.51±43.55
Total (N = 117)	37.14±7.71	25.72±12.37	4.01±3.10	11.15±8.60	184.32±55.05
*P* value	0.373	0.282	**0.001**	**<0.001**	0.445

Data are expressed as means ± SD. CSF = Cerebrospinal fluid; 25(OH)D = 25-hydroxyvitamin D.

### Correlation analysis of CSF and serum vitamin D levels

**Fig 1:**
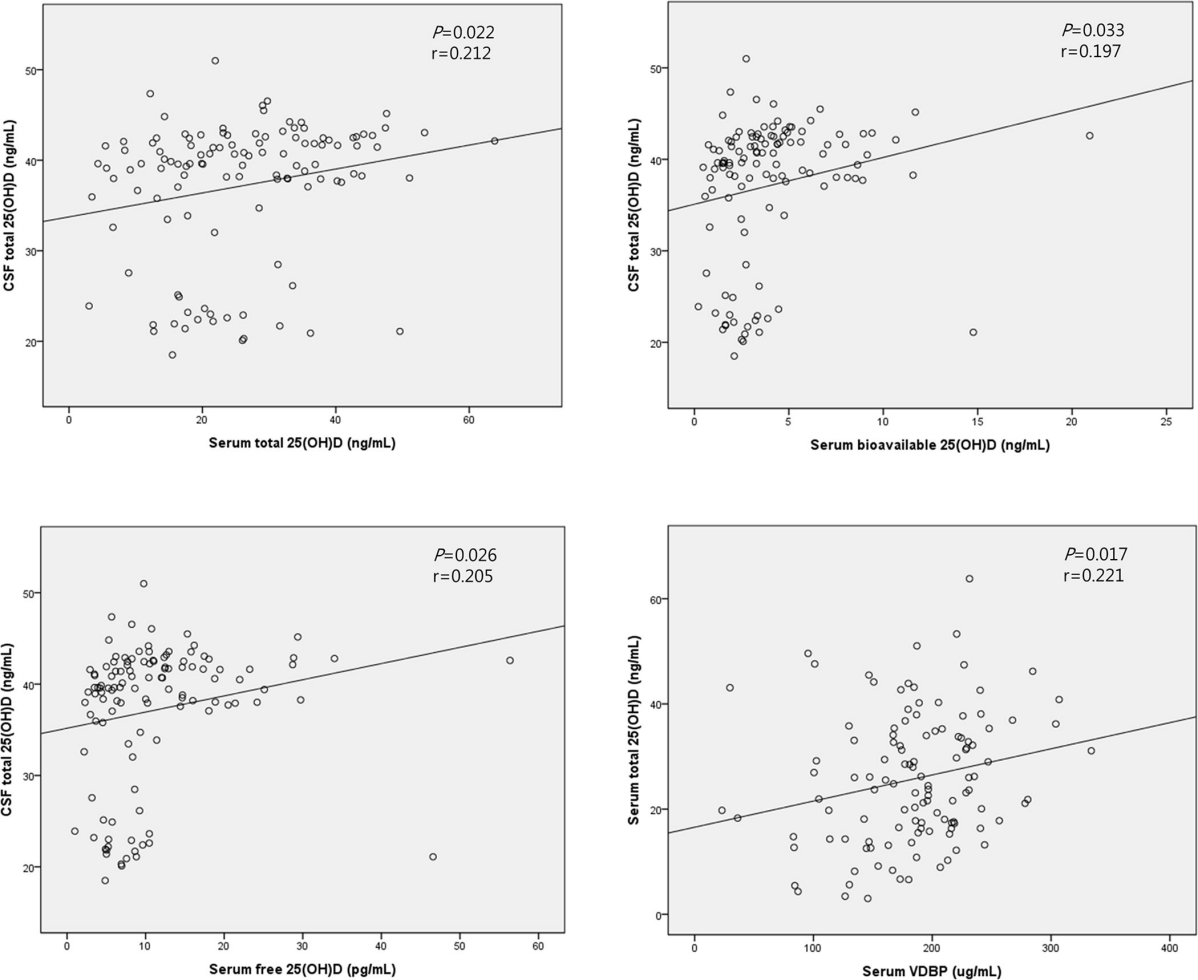
Correlation analysis of CSF and serum vitamin D levels. (r = Pearson correlation coefficient).

CSF total 25(OH)D concentrations in all patients showed significantly positive correlations with serum total, bioavailable, and free 25(OH)D concentrations (*P* = 0.022, *P* = 0.033, and *P* = 0.026, respectively). Serum total 25(OH)D concentration was also correlated with serum VDBP concentration (*P* = 0.017) (Fig 1). All r values in these correlations ranged from +0.1 to +0.3, implying weakly positive correlations.

### Distribution of CSF total 25(OH)D in neurologic and non-neurologic disease patients

**Fig 2:**
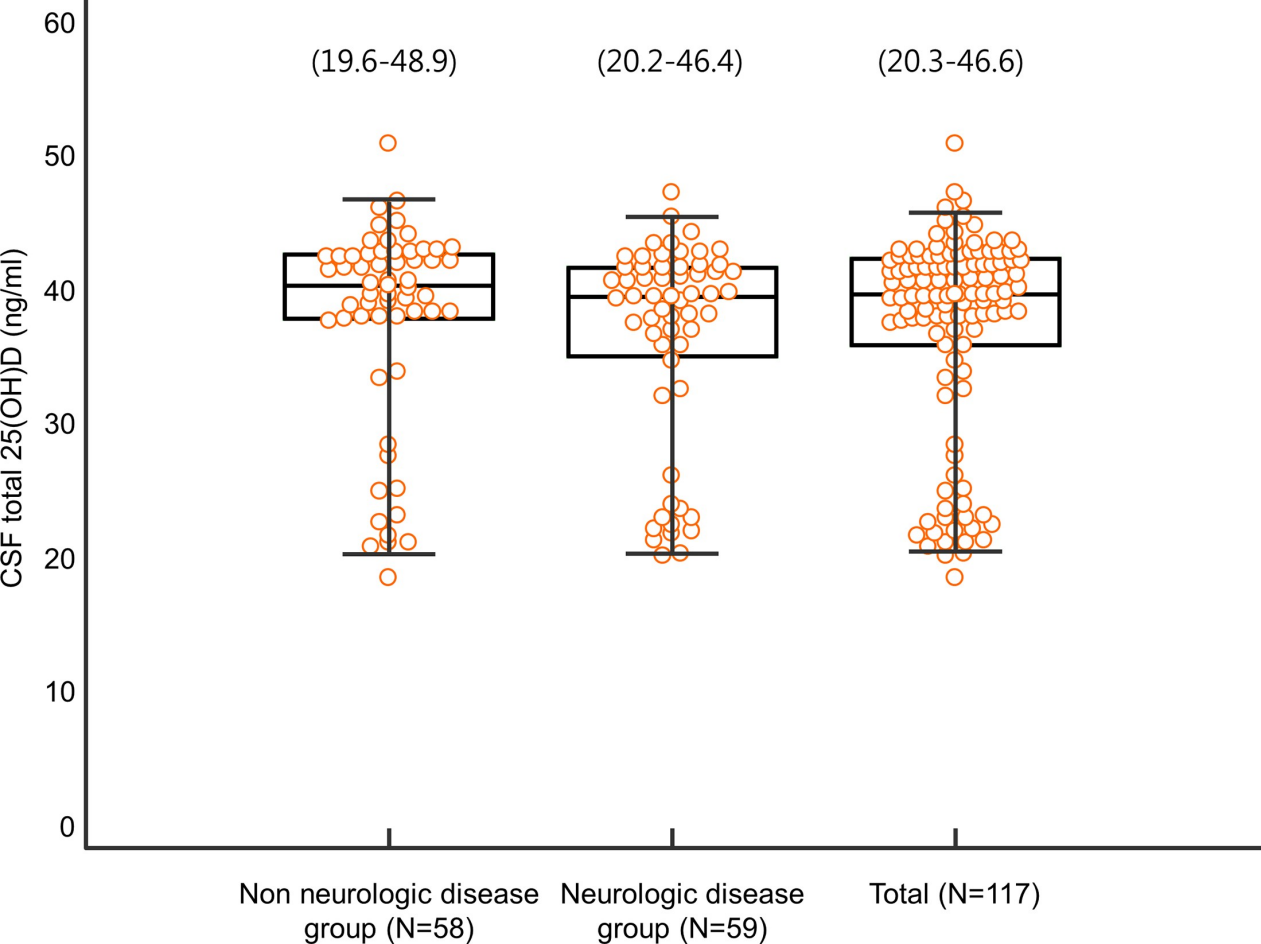
Distribution of CSF total 25(OH)D in neurologic and non-neurologic disease patients. The central box represents values from the lower to the upper quartile. The middle line represents the median. Bars represent the 2.5^th^ and 97.5^th^ percentiles.

We attempted to provide distribution of CSF total 25(OH)D in Korean neurologic and non-neurologic disease patients. Distributions of CSF total 25(OH)D levels in non-neurologic disease, neurologic disease, total patients in our study were 19.6–48.9, 20.2–46.4, 20.3–46.6 ng/mL, respectively. Percentile values (2.5th and 97.5th percentiles with median) of CSF total 25(OH)D concentration are shown in Fig 2.

## Discussion

Brain and general circulation are completely separated by the blood brain barrier (BBB) which tightly controls the transport of blood substances into the brain. Vitamin D is expected to be more difficult to pass through the BBB because most vitamin D are present in a very strongly bound form to VDBP. Recently, it has been reported that various neurodegenerative diseases are associated with serum or CSF vitamin D level [18–21,24]. However, little is known about the relationship between serum vitamin D and CSF vitamin D levels. Therefore, we designed this study to determine correlations between CSF and serum vitamin D levels. To the best of our knowledge, this is the first investigation to analyze correlations between CSF and serum vitamin D using clinical samples from patients. In this study, we demonstrated that total 25(OH)D level in CSF had weak positive but significant correlations with serum total, bioavailable, and free 25-hydroxy vitamin D levels in a Korean population. In addition, we presented the distribution of CSF total 25(OH)D in Korean neurologic and non-neurologic disease patients.

In our study, the mean CSF total 25(OH)D concentration was generally 1.4-fold higher than serum total 25(OH)D level. Such relatively high concentration of vitamin D in the brain may support a possible role of vitamin D in modulating inflammation and inducing regeneration of neuron suggested in a previous study [17]. In addition, our finding may imply that there is a special unidirectional transport system from general circulation to brain in BBB to maintain higher intrathecal vitamin D status. Most (85–90%) of circulating 25(OH)D in the serum are tightly bound to 58 kDa vitamin D binding protein (VDBP) while a smaller amount (10–15%) are loosely bound to albumin. Only less than 1% of circulating vitamin D exist in a free unbound form [4–6]. It has been reported that VDBP bound 25(OH)D, the most abundant form of vitamin D in serum, can be transported by megalin/cubilin transport system, the only transport system found for VDBP bound vitamin D [28]. Thus, this megalin/cubilin transport system might exist in BBB. To elucidate this, further studies including animal model investigations are needed.

There was no significant difference in CSF total 25(OH)D or serum VDBP concentration according to age group. However, there was a significant difference in serum total 25(OH)D concentration according to age group. Based on a recent study published by our group, serum total 25 (OH) D levels are the lowest in the 20s but the highest in the 70s [29]. In the present study, the age group of 18 to 65 years had lower serum total 25 (OH) D levels than the age group of over 65 years, consistent with our previous study [29]. In the present study, serum bioavailable 25(OH)D and serum free 25(OH)D levels were the lowest in the age group of 18 to 65 years but the highest in the age group of under 1 year. The present study is the first study to investigate serum bioavailable 25(OH)D and serum free 25(OH)D levels in almost all age groups. Similar to our findings, a previous study on young women has also revealed significant correlations of total 25(OH)D level with bioavailable 25(OH)D and free 25(OH)D levels [30].

According to disease group, there was no significant difference in any form of 25(OH)D between CSF and serum levels. Many studies have shown relationships of serum total 25(OH)D level with a variety of diseases. However, there are few studies about serum bioavailable and free 25(OH)D levels as markers of diseases. A recent study has shown that total and bioavailable 25(OH)D levels are significantly reduced in patients with nephrotic syndrome compared to those in healthy subjects [31]. That study suggested that bioavailable 25(OH)D level was a better standard measure in patients with nephrotic syndrome than total 25(OH)D level [31]. Multiple sclerosis is one of many diseases associated with vitamin D. Lower 25(OH)D levels are associated with higher disease activity. In addition, upregulated CSF vitamin D binding protein has been observed in those with multiple sclerosis. However, there is no significant association between disease activity and serum bioavailable 25(OH)D [32,33]. Another study has reported that CSF 25(OH)D levels are significantly lower in patients with Alzheimer's disease compared to those in normal subjects [24]. Our study did not find any association between various forms of serum vitamin D or CSF vitamin D and neurological diseases probably due to the small number of patients. Thus, further studies in large scale are needed to elucidate the association of serum and CSF vitamin D with any neurologic disease.

Although the relationship between various forms of serum vitamin D concentrations has been known so far, little is known regarding the relationship between serum and CSF 25(OH)D concentrations in human. In the present study, CSF total 25(OH)D showed weakly positive but significant correlations with serum total 25(OH)D, serum bioavailable 25(OH)D, and serum free 25(OH)D levels. This finding can be interpreted in relation to a special transport system of vitamin D in BBB [34]. Since 25(OH)D is tightly bound to VDBP which has a structure and size quite similar to albumin, access of 25(OH)D to the central nerve system and CSF is therefore restricted unless there are special transport mechanisms. Our data do not support active transport. However, our data suggest that CSF total 25(OH)D concentration correlates with serum 25(OH)D concentration. This concurs with results of injection studies in animals [14,15]. In our study, serum total, bioavailable, and free 25(OH)D levels all showed weakly positive but significant correlations with CSF total 25(OHD) level with similar r values. The reason why they only show weak correlations remains unknown. We may speculate that there is a unidirectional vitamin D transport system in BBB to maintain high concentration of intrathecal vitamin D which could affect correlation analysis. Further study is needed to elucidate this.

The major *GC* genotype and allele frequencies are known to vary among ethnicities [12]. For example, Nielson *et al*. [35] have reported that nearly all African American subjects and all Gambian subjects have Gc1f allele (Gc1f-Gc1f, Gc1f-Gc1s, or Gc1f-Gc2). In contrast, most white subjects do not have the Gc1f allele while Gc1s-Gc1s and Gc1s-Gc2 are the most frequent genotypes in this group [35]. Koreans have different GC allele frequencies than African Americans or whites. Jung *et al*. [36] have enrolled 203 patients with chronic obstructive pulmonary disease and 157 control subjects and reported that Gc1f-Gc2 (25%) is the most frequent genotype, followed by Gc1f-Gc1f (22%), Gc1f-Gc1s (20%), and Gc1s-Gc2 (18%), which is not exactly the same as our results, but similar in that the most frequent three genotypes have Gc1F allele. Serum bioavailable 25(OH)D and serum free 25(OH)D concentration showed significant differences according to genotype in our study. There results were expected since genotype results were used in the calculation of serum bioavailable and free 25(OH)D.

In our study, patients were divided into two groups, non-neurologic disease group and neurologic disease group, according to results of CSF analysis and final diagnosis. Patients in the non-neurologic group were patients with suspected neurologic disease for whom CSF analysis was performed without finding any particular neurological abnormalities. We also presented distribution of CSF total 25 (OH) D in patients with non-neurologic disease as well as those with neurologic disease. Ideally, providing a CSF total 25 (OH) D reference range for a healthy person would be a good baseline data. However, it might be impossible to suggest a reference range using CSF samples from healthy people. Instead, we presented distribution of CSF total 25 (OH) D in patients with non-neurologic disease in this study. These results could be valuable basic data for further vitamin D studies using CSF specimens.

This study has some limitations. First, this was a cross-sectional study. Second, although the disease group included various diseases, the number of patients included in each group was relatively small. Further research with larger scale for each group would be necessary to confirm findings of this study. Third, we did not investigate individual vitamin D supplementation, diet related information, or outdoor activity. It is well known in the literature that serum vitamin D levels are affected by these factors. Therefore, failure to investigate additional vitamin D related information is a limitation. Finally, we did not use liquid chromatography-tandem mass spectrometry (LC-MS/MS), a gold standard method, to measure total 25(OH)D levels. However, the Elecsys Vitamin D Total Kit with the Cobas e602 module (Roche Diagnostics, Mannheim, Germany) used in this study has been previously evaluated and reported to be comparable to LC-MS/MS method [37].

## Conclusions

Results of the present study demonstrate that total 25(OH)D level in CSF has weakly positive but significant correlations with serum total, bioavailable, and free 25-hydroxy vitamin D levels in a Korean population. In addition, we presented the distribution of CSF total 25(OH)D in Korean neurologic and non-neurologic disease patients. Results of our study on the relationship between serum vitamin D level and CSF vitamin D level can be used as basic data for future clinical studies and development of therapeutic drugs.
